# Single Surgeon Experience with 500 Cases of the Robotic Bilateral Axillary Breast Approach (BABA) for Thyroid Surgery Using the Da-Vinci Xi System

**DOI:** 10.3390/jcm10184048

**Published:** 2021-09-07

**Authors:** Yun-Suk Choi, Woo-Young Shin, Jin-Wook Yi

**Affiliations:** Department of Surgery, Inha University Hospital & College of Medicine, Incheon 22332, Korea; yunsukki@gmail.com (Y.-S.C.); mesik@hanmail.net (W.-Y.S.)

**Keywords:** thyroid neoplasm, thyroidectomy, robotic surgical procedures

## Abstract

Objectives: Robotic bilateral axillary breast approach (BABA) thyroid surgery began in 2008 and is now one of the most widely used remote-access thyroid surgeries worldwide. This study aimed to analyze the results of 500 robotic BABA thyroid surgeries performed in a single institution in Korea compared with open thyroid surgery. Methods: From December 2018 to March 2020, 502 robotic BABA thyroidectomies (RTs) and 531 conventional open thyroidectomies (OTs) were performed in our institution by a single endocrine surgeon. We retrospectively reviewed patient medical records and performed a comparative analysis of OT and RT. Results: The RT group was younger (43.41 ± 11.41 versus 54.28 ± 13.41 years, *p* < 0.001) and had a higher proportion of females (84.3% versus 69.3%, *p* < 0.001), a lower BMI (24.66 ± 3.97 versus 25.83 ± 4.07 kg/m^2^), a higher proportion of lobectomies (52.6% versus 45.2%) and a lower proportion of lateral neck dissections (3.4% versus 10.0%, *p* < 0.001). The RT group had a longer operation time (145.33 ± 40.80 versus 93.39 ± 43.55 min, *p* < 0.001) and higher surgical costs. Although the OT group had a larger tumor size and a higher proportion of extrathyroidal extension, the numbers of retrieved lymph nodes were not significantly different between the two groups. Additionally, there was no difference in the stimulated thyroglobulin level before radioactive iodine therapy (7.01 ± 35.73 versus 8.39 ± 58.77, *p* = 0.782). The rates of transient vocal cord palsy and transient hypoparathyroidism were significantly lower in the RT group, and those of scar-related complications were higher in the OT group. Conclusions: Robotic BABA thyroid surgery has advantages not only in better cosmetic outcomes but also in lower rates of vocal cord palsy and hypoparathyroidism, with comparable lymph node retrieval and serum thyroglobulin levels.

## 1. Introduction

The incidence of thyroid cancer has increased worldwide for reasons that are not yet clear. Although overdiagnosis is a problem, thyroid cancer is the 2nd most common cancer per the 2017 national statistics in Korea [1]. Thyroid cancer occurs frequently between the ages of 30 and 60, and the rate is three times higher among females [2,3]. Despite its high prevalence, thyroid cancer has a favorable survival rate, with a 10-year overall survival rate of 95%. Thus, aspects of quality of life after thyroid surgery, such as the cosmetic outcome and voice problems, and hypoparathyroidism are very important for thyroid cancer survivors. For this reason, many thyroid surgical approaches have been developed to not only reduce surgical scarring and preserve the nerve and parathyroids but also to achieve oncologic outcomes that are comparable to those of conventional surgical methods.

The anterior cervical approach using the transverse skin incision, also known as open thyroidectomy (OT), has been considered the standard method for thyroid surgery for over 100 years. However, OT inevitably leaves a visible and easily noticeable scar on the neck and causes many problems, including emotional and social stress from scar formation and other medical problems such as hypertrophic scarring, keloids, and straining or adhesions around the scar. To solve this problem, various remote-access thyroid surgical methods have been developed by pioneer surgeons worldwide. The first study on remote-access neck surgery was endoscopic parathyroidectomy performed by Gagner in 1996 [4]. Currently, the transaxillary approach (TA), bilateral axillary breast approach (BABA), retroauricular (RA) approach, and transoral approach (TO) are being actively implemented worldwide [5,6,7,8]. These methods were initially performed in an endoscopic manner, but the 2D vision and limited range of motion due to stiff and jointless endoscopic instruments made the surgery more difficult than OT. However, with the advancement of robotic surgical devices (the da-Vinci surgical system), remote-access thyroid surgical methods are shifting to robotic thyroidectomy because it can provide a better visual field through a 15-fold magnified full 3D vision and a wide range of motion by means of the endowrist function that implements the same movement as a surgeon’s hand [9,10,11].

The robotic BABA was initially started in 2008 at the Seoul National University Hospital, and the hospital recently reported more than 1000 cases of robotic BABA [12,13,14]. Regarding the robotic BABA, many studies have reported that robotic thyroidectomy is a good surgical method in terms of surgical stability and favorable surgical completeness [12,15,16,17]. Furthermore, robotic excellent surgical stability and completeness have also been reported in other part surgery [18]. Our hospital adopted the da-Vinci Xi system in December 2018 and began performing robotic BABA thyroid surgery with a single endocrine surgeon [19]. As of March 2020, more than 500 cases of robotic BABA thyroid surgery were performed in our institution, and this is the highest number of robotic thyroid surgeries in the city of Inchon in Korea. In this study, we compared our results of robotic BABA thyroid surgery with the OT results and evaluated the advantages and disadvantages of RT compared to OT.

## 2. Materials and Methods

From December 2018 to March 2020, a total of 502 robotic BABA thyroid surgeries were performed in our hospital. During the same period, a total of 531 thyroid surgeries were performed by the OT method. We retrospectively reviewed these patients’ electrical medical records, clinical characteristics, pathologic reports, laboratory findings, and postoperative complications. Robotic BABA patients were allocated to the “RT group”, and patients undergoing open surgery were allocated to the “OT group”. All surgeries were performed by a single endocrine surgeon. Indications for RT were not different from those for OT in our hospital. The selection of the surgical method was based on a thorough discussion with the surgeon and patients, with informed consent about the pros and cons of robotic thyroid surgery.

Thyroidectomy extent was decided based on the American Thyroid Association 2015 guidelines for both OT and RT. If papillary thyroid cancer (PTC) was suspected on fine-needle aspiration cytology (FNAC), prophylactic or therapeutic central neck lymph node dissection was performed according to the preoperative imaging study. When lateral neck lymph node metastasis was proven, modified radical neck dissection (MRND) was performed.

All patients who underwent total thyroidectomy were checked for serum thyroglobulin (Tg) levels 3 months after surgery. Patients who were considered at high risk of recurrence according to the 2015 guidelines of the American Thyroid Association underwent radioactive iodine (RAI) therapy. The TSH-stimulated Tg concentration in serum was measured on the day of RAI administration after levothyroxine withdrawal or recombinant TSH injection with dietary iodine restriction.

Vocal cord status was evaluated before and after surgery by laryngeal ultrasound or laryngoscopy. Permanent vocal cord palsy was defined as nonrecovery of vocal cord movement after 6 months. Transient hypoparathyroidism was defined as a serum intact parathyroid hormone (iPTH) level <5 pg/mL within 2 weeks after surgery, whereas permanent hypoparathyroidism was defined as a serum iPTH level <10 pg/mL 6 months after surgery. Patients who have clinical hypocalcemia symptoms that require oral calcium drug supplementation are also classified as having hypoparathyroidism, either transient or permanent. With the exception of lobectomy patients in the OT group, who were discharged on the 2nd day after surgery, patients in both the OT and RT groups were permitted to be discharged from the hospital generally on the 3rd day after surgery. We also reviewed the cost of surgery in US dollars, defined as the surgery fee but not the total medical cost, which would include hospitalization, because these costs vary among patients due to socioeconomic and health insurance-related factors.

All statistical analyses were performed using SPSS version 22.0 software (IBM Corp., Armonk, NY, USA). Continuous variables were compared by means of unpaired *t*-tests, and categorical variables were compared by means of chi-square tests. Regarding ethical considerations, our study was approved by the Institutional Review Board of our hospital (IRB number: INH-2021-04-044).

### 2.1. Open Thyroidectomy Procedure

For thyroidectomy procedures such as lobectomy and total thyroidectomy, a 5–7-cm low neck collar incision was used. Thyroid resection was performed in the following order: isthmus division, lateral mobilization with middle thyroid vein ligation, superior pole ligation, recurrent laryngeal nerve (RLN) and parathyroid identification, inferior thyroid vessel ligation, and berry ligament division. In the case of open MRND, a long transverse cervical incision of approximately 15 cm was made, followed by total thyroidectomy with central neck node dissection and lymph node dissection from level II to V and jugular lymph nodes.

### 2.2. Robotic BABA Thyroidectomy Procedure

Robotic BABA thyroid surgery was performed based on the method that we previously reported elsewhere [19]. Thyroid resection was performed in the following order: isthmus division, lateral mobilization with middle thyroid vein ligation, RLN, and parathyroid identification, inferior thyroid vessel ligation, berry ligament division, and superior pole dissection. In the case of robot BABA thyroidectomy with MRND, we also followed the same method as previously described elsewhere [9].

## 3. Results

Table 1 shows the clinical characteristics of the enrolled patients. In the RT group, the mean age of the patients was significantly lower (43.41 ± 11.41 versus 54.24 ± 13.41 years, *p* < 0.001), and the proportion of women were higher (84.3% versus 69.3%). The body mass index (BMI) was also significantly lower in the RT group (24.66 ± 3.97 versus 25.83 ± 4.07, *p* < 0.001). Among the preoperative FNAC results, the rates of Bethesda categories V and VI were higher in the RT group, whereas Bethesda categories II and III were higher in the OT group. There was no difference in tumor location between the two groups. The proportion of lobectomy was higher in the RT group, whereas that of total thyroidectomy was higher in the OT group. Regarding lymph node dissection, the rate of central node dissection (CND) was higher in the RT group (96.6% versus 90.0%, *p* < 0.001), and that of lateral neck dissection (LND) was higher in the OT group (10.0% versus 3.4%, *p* < 0.001). The operation time was significantly longer in the RT group (145.33 ± 40.80 versus 93.39 ± 43.55 min, *p* < 0.001), and the estimated blood loss (EBL) was also significantly higher in the RT group (56.29 ± 73.96 versus 41.46 ± 107.71 ml, *p* = 0.01). The hospital stay period was longer in the RT group, and the surgical costs were approximately 8.58 times higher in the RT group (USD 854.15 ± 361.04 vs. 7330.54 ± 462.84, *p* < 0.001). The number of patients who received RAI treatment after surgery was significantly higher in the OT group.

The pathologic findings after thyroidectomy are described in Table 2. The proportion of PTC was 79.5% (399/502) in the RT group and 69.5% (369/531) in the OT group. The proportions of benign and follicular tumors, including follicular adenoma (2/502 versus 1/531), nodular hyperplasia (2/502 versus 1/531), chronic lymphocytic thyroiditis (1/502 versus 2/531), follicular thyroid cancer (9/502 versus 3/532), Hürthle cell cancer (4/502 versus 1/531) and noninvasive follicular thyroid neoplasm with papillary-like nuclear features (NIFTP) (11/502 versus 6/531), were 18.9% (95 patients) in the RT group and 28.2% (150 patients) in the OT group. The proportion of PTC was significantly higher in the RT group, whereas that of benign/follicular tumors was higher in the OT group. The largest tumor sizes among PTC cases were 0.92 ± 0.71 cm in the RT group and 1.20 ± 0.99 cm in the OT group (*p* < 0.001). The largest tumor sizes among benign/follicular tumor cases were 2.86 ± 1.44 cm in the RT group and 4.38 ± 2.18 cm in the OT group (*p* < 0.001). The incidence of extrathyroidal extension, including the microscopic and gross extension in PTC, was significantly higher in the OT group. The number of retrieved nodes in the CND was not significantly different between the OT and RT groups (4.84 ± 4.46 versus 4.74 ± 4.18, *p* = 0.741). Harvested lymph nodes in the MRND group were also not different between the two groups (34.19 ± 14.73 versus 30.18 ± 11.59, *p* = 0.692). Additionally, the numbers of metastatic lymph nodes were not significantly different between the OT and RT groups or between the CND and MRND groups, as shown in Table 2. The proportion of *BRAF*^V600E^ mutations was higher in the OT group.

Values related to surgical completeness for PTC are described in Table 3. Serum thyroglobulin (Tg) levels measured at 3 months postoperatively were not different between the OT and RT groups (2.56 ± 32.27 versus 0.51 ± 2.75, *p* = 0.329). The proportions of patients with serum Tg levels below 1 ng/mL were 92.1% in the OT group and 93.3% in the RT group. A total of 165 patients in the OT group and 124 patients in the RT group received RAI. The 1st RAI dose was higher in the OT group (98.94 ± 41.13 versus 85.08 ± 35.14, *p* = 0.003). The TSH level before RAI administration was not different between the OT and RT groups (118.92 ± 59.24 versus 129.01 ± 43.40, *p* = 0.111) nor was the TSH-stimulated Tg level (7.02 ± 35.73 versus 8.39 ± 58.77, *p* = 0.782), and the proportion of patients with stimulated Tg levels below 1.0 did not differ according to the surgical approach method (46.1% in the OT group and 54.0% in the RT group, *p* = 0.180).

Table 4 shows the surgical complications in our patients. The transient vocal cord palsy rate was significantly lower in the RT group (3.8% versus 1.6%, *p* = 0.032). The permanent vocal cord palsy rate did not differ between the surgical approaches. The transient hypoparathyroidism rate was also significantly lower in the RT group (14.7% versus 8.4%, *p* = 0.004). Additionally, the rate of permanent hypoparathyroidism was not different between the two groups. Furthermore, the serum intact parathyroid hormone level after surgery was not significantly different between the OT and RT groups, but the rate of wound seroma was significantly higher in the RT group (4/532 (0.8%) versus 19/501 (3.8%), *p* < 0.001). The rates of scar-related complications, including keloids and hypertrophic scars, were significantly higher in the OT group (24/532 (4.5%) vs. 1/501 (0.2%), *p* < 0.001). Other complications, such as adhesion, bleeding, chyle leakage, Honor’s syndrome, and surgical site infection, were reported at low numbers, as shown in Table 4.

## 4. Discussion

The first endoscopic BABA thyroidectomy was performed in 2004, and the first robot BABA thyroidectomy was performed in 2008 at Seoul National University in Korea [15,20]. Unlike other endoscopic or robotic thyroidectomy methods, such as the transaxillary or retroauricular approaches, the BABA provides a symmetric, surgical view of both thyroid lobes, which is similar to conventional open thyroidectomy. This enables optimal visualization of important structures such as the parathyroid gland, recurrent and superior laryngeal nerves, and blood vessels [21]. In the early time period of BABA RT, surgical indications for thyroid cancer were very narrow and included thyroid cancer measuring under 1 cm, no evidence of lymph node metastasis, no evidence of extrathyroidal extension, concurrent thyroiditis or Graves’ disease, and no history of neck radiation, among others [10,21]. BABA RT has been performed for more than 10 years, and its surgical indications expanded widely to include large thyroid cancers, minor extrathyroidal extension, neck node dissection including central and lateral neck nodes (MRND), and combined thyroiditis or Graves’ disease [9]. Surgical and oncologic safety has also been shown in many previous studies [4,12,17,21,22].

Beginning on December 24, 2018, BABA RT using the da-Vinci Xi system was started in our hospital. More than 200 cases have been performed per year, and a total of 500 cases have been performed within 3 years by a single endocrine surgeon. As of May 2021, this is the world’s largest number of BABA RT procedures, performed by only the 4th generation model da-Vinci Xi system. The advantages of the Xi system compared to the da-Vinci Si system are as follows: It uses only 8-mm trocars which were reduced from the 12-mm camera port in the da-Vinci Si system. Camera access is possible with 4 ports, so we can get various viewpoints and provides new endowrist instruments such as Vessel Sealer Extend [23]. Thus, we can reduce the breast and axillary scar size and expand our surgery to MRND cases, as we reported elsewhere [9,19].

Although the patients were older, the proportion of males was higher, the tumor size was larger, and the BMI was higher in our analysis, the surgical indication for BABA RT in our hospital was not different from that for OT. Contraindications of BABA RT in our hospital are a direct invasion of adjacent organs by the main tumor or metastatic nodes (trachea, vertebra, carotid artery, jugular vein, etc.), a previous history of open thyroid or neck surgery, and having a high risk of an underlying medical condition. The decision to perform BABA RT was based on patient preference after receiving a full explanation of the RT procedure, including the pros and cons, compared to open surgery. Informed consent for BABA RT, including knowledge of the surgical advantages and disadvantages and the high cost of robotic surgery, was obtained from patients by the medical staff in our hospital.

To evaluate oncologic issues for thyroid cancer surgery, the number of harvested lymph nodes and surgical completeness using the serum thyroglobulin level should be evaluated [13,22]. In our patients who underwent CND and MRND, there was no significant difference in the harvested lymph nodes between the OT and RT groups, as shown in Table 2. This finding means that lymph node dissection for thyroid cancer can be effectively achieved by the BABA RT method. Serum Tg is used to evaluate recurrence or residual disease in patients undergoing total thyroidectomy, and TSH-stimulated Tg is an index that directly reflects the amount of remnant thyroid tissue after surgery. In our study, the proportion of patients with serum Tg less than 1 ng/mL at 3 months was over 92% in both groups, and there was no significant difference. The proportion of stimulated Tg levels before RAI below 1 ng/mL was also not different between OT and RT. According to our results, the surgical completeness of BABA RT is not inferior to that of OT. Specifically, the number of RAI-treated patients was higher in the OT group, and the RAI dose was also higher. It is thought that more patients with relatively severe thyroid cancer were included in the OT group than in the RT group. Despite this selection bias, we can suggest that RT is comparable to OT in that it can be used to completely remove the thyroid tissue and regional lymph nodes.

Regarding surgical complications according to thyroid surgery, vocal cord palsy and hypocalcemia are important issues for surgical safety. Our RT group had a significantly lower incidence of transient vocal cord palsy than the OT group. This result may be due to the 15-fold magnified view in the robotic device, fine dissection around the nerve, and routine use of intraoperative neuromonitoring in BABA RT, although the effect of neuromonitoring should be further evaluated in future research. The incidence of transient hypocalcemia was significantly lower in the RT group than in the OT group. This phenomenon may also be due to advantages in terms of the robotic camera vision and the fine surgical devices of the robotic arms. Higher visibility reveals the detailed vascular structure around the parathyroid glands, and precise robotic surgical instruments can be used more precisely to preserve parathyroid tissue and surrounding vessels. More studies are needed to establish a clinical basis for the preservation of the parathyroid gland using robotic surgery.

Neck wound hypertrophic scars or keloids occurred significantly more often in the OT group than in the RT group and seroma occurred more frequently in the RT group due to a large flap from the breast and axilla to the thyroid. Seromas were completely resolved by simple aspiration at the outpatient clinic and did not result in sequelae among the patients. Regarding other minor complications, there were no differences in bleeding, adhesions, chyle leaks, or infections between the two groups. The number of hospital stay days was higher in the RT group in our analysis because patients who received open thyroid lobectomy were permitted to be discharged from the hospital on the 2nd postoperative day. Other patients, including those with open total thyroidectomy, robotic lobectomy, and robotic total thyroidectomy, were permitted to be discharged after the 3rd postoperative day. Thus, the hospital stay was approximately 0.5 days longer in the RT group. The surgical time was approximately 50–55 min longer in the RT group than in the OT group, and the flap time for RT surgery was approximately 30 min. When the console time excluding flap time in the RT group was compared with the operation time of the OT group, the RT group operation time was significantly longer. (115.56 ± 38.21 versus 93.39 ± 43.55, *p* < 0.001) EBL was also higher in the RT group; however, the difference between groups was only 15–20 mL, and it did not affect the clinical outcome of patients in the RT group.

Since open thyroidectomy leaves a long scar in a prominent location of the neck, young female patients may experience social discomfort after surgery [24]. Robot thyroidectomy is considered to provide the greatest benefit in terms of hiding this wound in an invisible place and reducing the scar problem by making the scar size smaller. However, the biggest weakness is its high cost [25]. The cost was found to be 8.58 times that of OT in our study. When the options are explained to the patient before surgery, it is necessary to note the patient’s cancer status and the exact cost of treatment and to select the RT targets by considering the patient’s personal insurance status and socioeconomic status.

Since this study is a retrospective study, there may be a problem due to selection bias, as mentioned earlier. In addition, since the results are based on procedures performed by a single experienced surgeon in a high-volume center over 500 cases of thyroid surgery annually, other research will be needed to confirm these results in a small-volume center or with a novice surgeon. Since this study was based only on short-term follow-up data, additional research on long-term follow-up data is needed through follow-up observations of more than 10 years.

## 5. Conclusions

Since the beginning of robotic BABA surgery more than 10 years ago, our hospital has adopted robotic BABA thyroid surgery using the da-Vinci Xi system and successfully performed more than 500 cases within 3 years. Our report showed better surgical safety, lower incidences of transient vocal cord palsy and hypoparathyroidism, and comparable surgical completeness. Due to the relatively short-term follow-up period, a long-term observational study is required.

## Figures and Tables

**Table 1:** Clinical characteristics and pathologic variables of the patients included in this study.

Variable	Total	OT (*n* = 531)	RT (*n* = 502)	*p*-Value
Age (years, mean ± SD)	48.99 ± 13.61	54.28 ± 13.41	43.41 ± 11.41	<0.001
	(35.38–62.60)	(40.87–67.69)	(32.00–54.82)	
Sex				
Male	242 (23.4%)	163 (30.7%)	79 (15.7%)	<0.001
Female	791 (76.6%)	368 (69.3%)	423 (84.3%)	
BMI (Body mass index, kg/m^2^)	25.29 ± 4.07	25.83 ± 4.07	24.66 ± 3.97	<0.001
Fine-needle aspiration cytology (Bethesda category)				
Papillary thyroid cancer (VI)	543 (52.6%)	259 (49.8%)	284 (56.7%)	<0.001
Suspicious of papillary thyroid cancer (V)	173 (16.7%)	79 (14.9%)	94 (18.7%)	
Follicular neoplasm or suspicion for a follicular neoplasm (IV)	58 (5.6%)	25 (4.7%)	33 (6.6%)	
Atypia of undermined significance (III)	67 (6.5%)	41 (7.7%)	26 (5.2%)	
Benign (II)	172 (16.7%)	117 (22.0%)	55 (11.0%)	
Nondiagnostic/Inadequate (I)	8 (0.8%)	3 (0.6%)	5 (1.0%)	
For completion thyroidectomy	12 (1.2%)	7 (1.3%)	5 (1.0%)	
Tumor location				
Right	416 (40.3%)	205 (38.6%)	211 (42.0%)	0.665
Left	469 (45.4%)	243 (45.8%)	226 (45.0%)	
Bilateral	138 (13.4%)	75 (14.7%)	60 (12.0%)	
Isthmus	10 (1.0%)	5 (0.9%)	5 (1.0%)	
Thyroidectomy extent				
Lobectomy	504 (48.8%)	240 (45.2%)	264 (52.6%)	<0.001
Total thyroidectomy	494 (47.8%)	264 (49.7%)	230 (45.8%)	
Completion	35 (3.4%)	27 (5.1%)	8 (1.6%)	
Lymph node dissection				
Central neck dissection	963 (93.2%)	478 (90.0%)	485 (96.6%)	<0.001
Lateral neck dissection	70 (6.8%)	53 (10.0%)	17 (3.4%)	
Operation time (min)				
All		93.39 ± 43.55	145.33 ± 40.80	<0.001
Lobectomy		74.96 ± 20.33	129.48 ± 28.49	<0.001
Total thyroidectomy		93.34 ± 27.72	156.29 ± 32.92	<0.001
Flap time			29.76 ± 9.23	
Console time			115.56 ± 38.21	
Estimated blood loss (mL, mean ± SD)		41.46 ± 107.71	56.29 ± 73.96	0.01
Hospital stay days after surgery (days, mean ± SD)		2.98 ± 1.08	3.48 ± 0.97	<0.001
Surgery costs (US dollar)		854.15 ± 361.04	7330.54 ± 462.84	<0.001
Radioactive iodine therapy after total thyroidectomy (numbers)				
No	744 (72.0%)	366 (68.9%)	378 (75.3%)	0.023
Yes	289 (28.0%)	165 (31.1%)	124 (24.7%)	

**Table 2:** Pathological findings.

Variable	OT (*n* = 531)	RT (*n* = 502)	*p*-Value
Pathologic diagnosis			
PTC *	369 (69.5%)	399 (79.5%)	0.001
Benign/Follicular tumors ^†^	150 (28.2%)	95 (18.9%)	
Others ^‡^	12 (2.3%)	8 (1.6%)	
Tumor size (cm, mean ± SD)	2.05 ± 1.99	1.28 ± 1.18	<0.001
PTC *	1.20 ± 0.99	0.92 ± 0.71	<0.001
Benign/Follicular tumors ^†^	4.38 ± 2.18	2.86 ± 1.44	<0.001
Extrathyroidal extension ^¶^			
Absent	242 (65.2%)	324 (76.2%)	<0.001
Present	129 (34.8%)	101 (23.8%)	
Lymph nodes, retrieved			
Central node dissection (CND)	4.84 ± 4.46	4.74 ± 4.18	0.741
Modified radical neck dissection (MRND)	34.19 ± 14.73	30.18 ± 11.59	0.692
Lymph nodes, metastatic			
Central node dissection (CND)	1.06 ± 2.01	1.08 ± 2.10	0.898
Modified radical neck dissection (MRND)	10.10 ± 7.42	9.29 ± 6.50	0.310
*BRAF*^V600E^ mutation			
Absent	294 (81.2%)	360 (86.7%)	<0.001
Present	68 (18.8%)	57 (13.4%)	

* Papillary thyroid cancers, including follicular variants, tall cell variants, solid variants, etc. ^†^ Follicular adenomas, nodular hyperplasias, chronic lymphocytic thyroiditis, follicular thyroid cancer, Hürthle cell cancer, and noninvasive follicular thyroid neoplasm with papillary-like nuclear features (NIFTP). ^‡^ Medullary thyroid cancer, poorly differentiated thyroid cancer, anaplastic thyroid cancer, etc. ^¶^ Including either microscopic and gross extrathyroidal extension.

**Table 3:** Surgical completeness in total thyroidectomy patients.

Variable	OT (*n* = 291)	RT (*n* = 238)	*p*-Value
Postoperative 3 months Tg * (mean ± SD, ng/mL)	2.56 ± 32.27	0.51 ± 2.75	0.329
<1 (number of patients, %)	268 (92.1%)	222 (93.3%)	0.605
>1 (number of patients, %)	23 (7.9%)	16 (6.7%)	
Number of patients who received RAI ^†^ therapy	165/531 (31.1%)	124/502 (24.7%)	0.023
1st RAI dose (mean ± SD, mCi)	98.94 ± 41.13	85.08 ± 35.14	0.003
TSH level before RAI (mean ± SD, uIU/mL)	118.92 ± 59.24	129.01 ± 43.40	0.111
Stimulated Tg level before RAI (mean ± SD, ng/mL)	7.01 ± 35.73	8.39 ± 58.77	0.782
<1 (number of patients, %)	76 (46.1%)	67 (54.0%)	0.180
>1 (number of patients, %)	89 (53.9%)	57 (46.0%)	

* Tg: thyroglobulin ^†^ RAI: radioactive iodine.

**Table 4:** Postoperative complications.

Variable	OT (*n* = 531)	RT (*n* = 502)	*p*-Value
Nerves at risk	795	732	N/A
Vocal cord palsy			
Transient (<6 months)	20 (3.8%)	8 (1.6%)	0.032
Permanent (>6 months)	4 (0.8%)	1 (0.2%)	0.2
Hypoparathyroidism			
Transient (<6 months)	78 (14.7%)	42 (8.4%)	0.004
Permanent (>6 months)	10 (1.9%)	12 (2.4%)	0.051
PTH * (pg/mL)			
<2 weeks	16.59 ± 16.80	17.28 ± 13.55	0.608
>3 months	26.61 ± 20.65	30.56 ± 28.17	0.066
Other complications			
Adhesion	0 (0.0%)	2 (0.4%)	<0.001
Bleeding	2 (0.4%)	3 (0.6%)	
Chyle leak	3 (0.6%)	2 (0.4%)	
Honor syndrome	0 (0.0%)	1 (0.2%)	
Infection	1 (0.2%)	0 (0.0%)	
Seroma	4 (0.8%)	19 (3.8%)	
Scar related (Kelloid/Hypertrophic)	24 (4.5%)	1 (0.2%)	
None	497 (93.6%)	474 (94.4%)	

* PTH: parathyroid hormone.

## Data Availability

No new data were created or analyzed in this study. Data sharing is not applicable to this article.
